# Ser^71^ Phosphorylation Inhibits Actin-Binding of Profilin-1 and Its Apoptosis-Sensitizing Activity

**DOI:** 10.3389/fcell.2021.692269

**Published:** 2021-06-21

**Authors:** Faliang Wang, Cuige Zhu, Shirong Cai, Aaron Boudreau, Sun-Joong Kim, Mina Bissell, Jieya Shao

**Affiliations:** ^1^Division of Oncology, Department of Medicine, Washington University School of Medicine, St. Louis, MO, United States; ^2^Department of Surgical Oncology, The Children’s Hospital, Zhejiang University School of Medicine, National Clinical Research Center for Child Health, Hangzhou, China; ^3^Department of Cell Biology and Physiology, Washington University School of Medicine, St. Louis, MO, United States; ^4^Department of Cancer Biology, The University of Texas MD Anderson Cancer Center, Houston, TX, United States; ^5^Life Sciences Division, Lawrence Berkeley National Laboratory, Berkeley, CA, United States

**Keywords:** profilin-1, phosphorylation, actin, poly-L-proline, apoptosis, breast cancer, chemotherapy, protein kinase A

## Abstract

The essential actin-binding factor profilin-1 (Pfn1) is a non-classical tumor suppressor with the abilities toboth inhibit cellular proliferation and augment chemotherapy-induced apoptosis. Besides actin, Pfn1 interacts with proteins harboring the poly-L-proline (PLP) motifs. Our recent work demonstrated that both nuclear localization and PLP-binding are required for tumor growth inhibition by Pfn1, and this is at least partially due to Pfn1 association with the PLP-containing ENL protein in the Super Elongation Complex (SEC) and the transcriptional inhibition of pro-cancer genes. In this paper, by identifying a phosphorylation event of Pfn1 at Ser^71^ capable of inhibiting its actin-binding and nuclear export, we provide *in vitro* and *in vivo* evidence that chemotherapy-induced apoptotic sensitization by Pfn1 requires its cytoplasmic localization and actin-binding. With regard to tumor growth inhibition byPfn1, our data indicate a requirement for dynamic actin association and dissociation rendered by reversible Ser^71^phosphorylation and dephosphorylation. Furthermore, genetic and pharmacological experiments showed that Ser^71^ of Pfn1 can be phosphorylated by protein kinase A (PKA). Taken together, our data provide novel mechanistic insights into the multifaceted anticancer activities of Pfn1 and how they are spatially-defined in the cell and differentially regulated by ligand-binding.

## Introduction

As the first actin-binding protein identified more than four decades ago (Carlsson et al., 1977), profilin-1 (Pfn1) has been extensively studied in the context of actin regulation. By binding monomeric G-actin, Pfn1 exchanges ADP for ATP and facilitates the addition of ATP-bound G-actin to the barbed ends of filamentous actin (Haarer and Brown, 1990; Witke, 2004; Jockusch et al., 2007; Birbach, 2008). In addition, Pfn1 interacts with a wide range of poly-L-proline (PLP)-containing proteins many of which are actin-regulatory factors and cooperate with Pfn1 to control actin polymerization (Haarer and Brown, 1990; Witke, 2004; Jockusch et al., 2007; Birbach, 2008). Pfn1 is essential for the development and survival of multiple eukaryotic organisms including mice, Drosophila and yeast (Balasubramanian et al., 1994; Verheyen and Cooley, 1994; Witke et al., 2001; Bottcher et al., 2009). Paradoxically, Pfn1 also shows anti-tumor and anti-metastatic activities for various types of cancer (breast, pancreatic, and liver) (Janke et al., 2000; Roy and Jacobson, 2004; Wittenmayer et al., 2004; Ding et al., 2006; Wu et al., 2006; Zou et al., 2007, 2009, 2010; Bae et al., 2009, 2010; Das et al., 2009; Yao et al., 2014; Diamond et al., 2015). Our prior study suggested that some of these anticancer activities may stem from nuclear Pfn1 (Diamond et al., 2015). Our more recent work supported this theory and demonstrated that nuclear Pfn1 functions as a transcriptional repressor by binding and inhibiting the Super Elongation Complex (SEC), a positive regulator of transcriptional elongation of many pro-cancer genes (Zhu et al., 2021). Furthermore, we provided evidence that Pfn1 undergoes spatial deregulation in a broad range of cancer due to overexpression of its nuclear exporter exportin-6. This explains, to some extent, how anticancer activity of nuclear Pfn1 can be inhibited while its essential cytoplasmic functions are sustained. However, given that Pfn1 influences distinct cancer phenotypes including proliferation, metastasis, and survival upon chemotherapy treatments (Zou et al., 2010; Yao et al., 2013; Zaidi et al., 2016), it remains unclear whether these activities stem from the same or different subcellular locations and whether there are additional regulatory mechanisms besides exportin-6-dependent nuclear export.

We have found in prior and recent studies that PLP-binding is important for the tumor-inhibitory function of Pfn1 (Diamond et al., 2015), at least partially due to the direct interaction of nuclear Pfn1 with ENL, a PLP-containing protein, in the SEC complex (Zhu et al., 2021). We and others found that PLP-binding of Pfn1 can be abolished by Ser^137^ phosphorylation in its C-terminus (Shao et al., 2008b; Diamond et al., 2015). In addition to PLP-binding, actin-binding was also suggested to be important for tumor inhibition by Pfn1. This was based on the loss-of-function effect of Y59A, an actin-binding mutation of Pfn1 (Schluter et al., 1998; Wittenmayer et al., 2004). However, despite being the first actin-binding protein identified several decades ago, it remains unknown whether phosphorylation events exist in Pfn1 which can inhibit its actin-binding and modulate its anticancer activities.

In this paper, we identified a protein kinase A (PKA)-dependent phosphorylation site in Pfn1 at Ser^71^. Residing in the actin-binding site of Pfn1, Ser^71^ phosphorylation abolishes the Pfn1/actin interaction and causes nuclear retention of Pfn1. Functional characterization using breast cancer cell lines revealed that Ser^71^ phosphorylation regulates both cell proliferation and chemotherapy-induced apoptosis but in different fashions. Dissection of the functional influences of subcellular localization further indicated that while tumor inhibition by Pfn1 is driven largely by its nuclear activities, apoptosis-sensitizing effect depends on its cytoplasmic localization. Thus, by identifying and characterizing a previously unknown inhibitory phosphorylation event for actin-binding of Pfn1, we provided further mechanistic insights into its multifaceted tumor-inhibitory activities which are regulated both by ligand-binding and subcellular localization.

## Materials and Methods

### DNA Constructs

Untagged, Myc-tagged, and HA-tagged Pfn1 in pcDNA3, His-tagged Pfn1 in pRK172, untagged Pfn1 in pLenti-CMV/TO-Neo-DEST, and YFP-Pfn1 with and without NES or NLS tag in pFLRu-FH vector have been described previously (Shao et al., 2008b; Diamond et al., 2015; Zhu et al., 2021). Point mutations (S71A and S71D) within Pfn1 were introduced by site-directed mutagenesis using QuikChange. GFP-tagged PKA catalytic subunit in EGFP-C1 was purchased from Addgene (plasmid # 61091).

### Antibodies

Primary antibodies used for Western blots are as follows: mouse anti-HA-tag (Convance, MMS-101P through BioLegend, United States), mouse anti-Myc-tag (Santa Cruz, United States, sc-40), mouse anti-β-actin (Santa Cruz, United States, sc-47778; Cell Signaling, United States, #3700), mouse anti-α-tubulin (Cell Signaling, United States, #3873), mouse GAPDH (Santa Cruz, United States, sc-47724), rabbit anti-Pfn1 (Cell Signaling, United States, #3246), rabbit anti-VASP (Bethyl laboratories, United States, A304-769A), rabbit anti-cleaved caspase-7 (Cell Signaling, United States, #8438), rabbit anti-cleaved PARP (Cell signaling, United States, #9541), rabbit anti-GFP (Cell Signaling, United States, #2956). To raise the polyclonal pSer^71^-Pfn1 antibody (F5675), a synthetic phospho-Pfn1 peptide harboring pSer^71^ [Ac-CLGGQKC(pS)VIRDSL-amide] was conjugated to keyhole limpet hemocyanin and used to immunize rabbits. Antiserum was subjected to double affinity purification using both the antigenic phospho-peptide and the same peptide without the phosphate on Ser^71^ (New England Peptide, Inc., United States).

### Cell Culture

All cell lines were purchased from ATCC with the exception of MDA-MB-231 cells stably expressing a tri-modal reporter fusion used to inject NOD/SCID mice as previously described (Diamond et al., 2015). All cell lines were authenticated and tested for mycoplasma within 3 months prior to the experiments. MDA-MB-231, MCF-7, and BT-549 were grown in RPM1 1,640 containing 5 or 10% fetal bovine serum (FBS) with gentamicin and supplements (50 μg/mL gentamycin, 1mM sodium pyruvate, 10 mM HEPES and glucose to 4.5 g/L). HEK293T cells were grown in high glucose DMEM supplemented with 5 or 10% fetal bovine serum and 50 μg/mL gentamicin. Transient transfection was performed using Fugene HD or Lipofectamine 2000. Lentiviruses were generated using HEK293T cells as previously described (Diamond et al., 2015).

### Pull-Down Assays

To study Pfn1 interaction with actin and VASP, HEK293T cells grown in 6-well dishes were transfected with Myc-tagged or HA-tagged Pfn1 constructs (WT and mutants), lysed by RIPA buffer, and subjected to immunoprecipitation using antibodies against Myc or HA tags as described previously (Shao et al., 2008b; Diamond et al., 2015). To affinity purify endogenous Pfn1, parental MDA-MB-231 cells were lysed and bound to PLP-conjugated agarose beads as described previously (Shao et al., 2008b; Diamond et al., 2015).

### *In Vitro* Drug Treatment

For drug treatment in 2D cultures, MDA-MB-231 or BT-549 stable cells were seeded at 1,000 cells per well in 96-well plates or 500 cells per well in 24-well plates, and treated on the next day with vehicle or paclitaxel in quadruplicate wells. Viable cells were quantified 5–7 days later by the Alamar Blue assay (Diamond et al., 2015; Zhu et al., 2021). Briefly, they were incubated with 100ul (for 96-well) or 500ul (for 24-well) growth media containing 44 μM resazurin for 2–4 h at 37°C, and the fluorescence intensity of resorufin (converted product) in the media was measured at 540λ Ex/590λ Em on a fluorescence plate reader (Tecan Infinite M200). Relative drug effects were calculated as the percentage of live cells in drug vs. vehicle wells.

### Mouse Xenografts

The animal experiment was carried out in strict accordance with the guidelines recommended for care and use of laboratory animals by the National Institutes of Health. The Animal Studies Committee at Washington University (St. Louis, MO, United States) approved all animal protocols. Five-week old female NOD/SCID and NU/NU mice were purchased from Charles River and kept under standard institutional care. Experimental details for orthotopically inoculating MDA-MB-231 stable cells were previously described (Diamond et al., 2015; Zhu et al., 2021). For paclitaxel dosing, when tumors in the NU/NU mice reached an average of ∼70 mm^3^, they were randomly divided and treated with 0.9% sodium chloride or paclitaxel (10 mg/kg) by weekly intraperitoneal injection for two consecutive weeks. Clinical grade paclitaxel (6 mg/ml) was purchased from Siteman Cancer Center pharmacy at Washington University School of Medicine. Primary tumors were measured by Caliper on a weekly (for NOD/SCID) and semiweekly (NU/NU) basis until tumor resection and euthanasia of the mice.

### Statistical Analysis

Unpaired two-tailed student *t*-test was used to determine the statistical significance of the differences in cell growth rates, tumor sizes and weights between control and experimental groups. All statistical analyses were performed using GraphPad Prism 7.0. *P-*values < 0.05 were considered significant.

## Results

### Ser^71^ Phosphorylation Abolishes Actin-Binding of Pfn1

**FIGURE 1:**
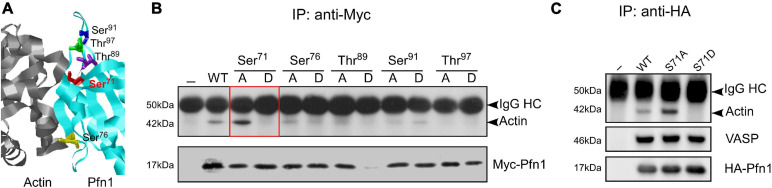
Mimicking Ser^71^ phosphorylation by the S71D mutation abolishes actin-binding of Pfn1. **(A)** X-ray diffraction structure of human Pfn1 (right, cyan) bound with actin (left, purple) (Protein Data Bank ID 2PAV) (Ferron et al., 2007). Ser^71^ and four other serine and threonine residues in the actin-binding region of Pfn1 are displayed in the sticks mode. **(B)** HEK293T cells were transfected with Myc-tagged wild type or mutant Pfn1 containing alanine (A) or aspartate (D) substitutions on the five candidate serine or threonine residues. Lysates were subjected to immunoprecipitation using a Myc-tag antibody, and Western blot was used to detect Myc-Pfn1 and co-precipitated actin. **(C)** HEK293T cells were transfected with wild type or mutant forms of HA-tagged Pfn1 containing S71A or S71D, which were subsequently immunoprecipitated by an anti-HA tag antibody. Lysates were subjected to immunoprecipitation, and samples were blotted for actin, VASP, and HA-tag (to visualize HA-Pfn1).

Hypothesizing that actin-binding of Pfn1 can be inhibited by phosphorylation, we selected five candidate serine/threonine residues at the actin-binding region of Pfn1 guided by the actin/Pfn1 co-crystal structure (Figure 1A; Ferron et al., 2007). Each residue was mutated to alanine (S/A) or aspartate (S/D) to prevent or mimic phosphorylation. The resulting Myc-tagged Pfn1 mutants were transfected into HEK293T cells and immunoprecipitated using an anti-Myc antibody followed by Western blot to determine their actin-binding. All mutants were successfully expressed except T89D which was likely due to a destabilizing effect on Pfn1 structure and its consequent degradation. Out of the five candidate residues, Ser^71^ appears to be a bona fide phosphorylation site as preventing (S71A) and mimicking (S71D) phosphorylation had opposite effects on actin-binding of Pfn1. While Pfn1(S71A) binds more actin than Pfn1(WT), Pfn1(S71D) completely fails to bind actin (Figure 1B). Introducing the S71A and S71D mutations into an HA-tagged Pfn1 showed the same effects on actin-binding as for Myc-Pfn1, but neither mutation had detectable effect on Pfn1 interaction with vasodilator-stimulated phosphoprotein (VASP), a well-known PLP-containing ligand of Pfn1 (Reinhard et al., 1995; Kang et al., 1997; Ferron et al., 2007; Figure 1C).

**FIGURE 2:**
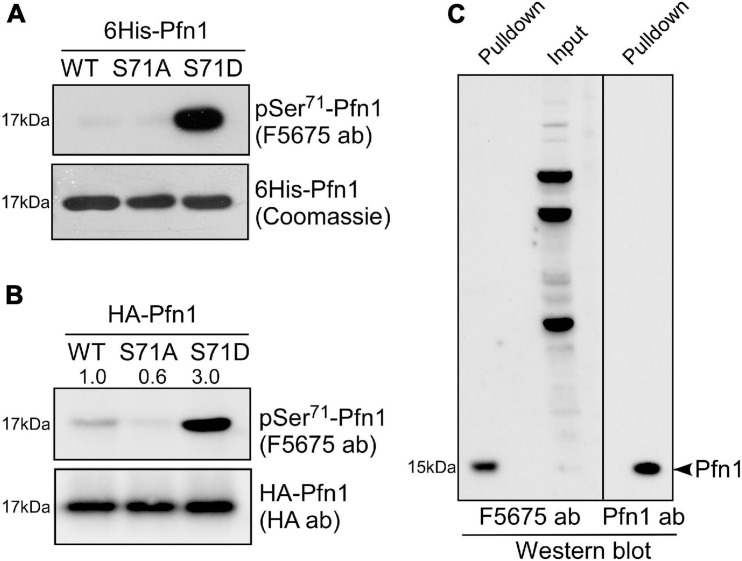
Pfn1 is phosphorylated at Ser^71^ in cultured cells. **(A)** Equal amounts of recombinant 6His-Pfn1 (WT, S71A, or S71) purified from bacteria was stained by Coomassie blue or analyzed by Western blot using the pSer^71^-Pfn1 antibody F5675. **(B)** HA-tagged Pfn1 (WT, S71A, or S71D) was transfected into HEK293 cells and immunoprecipitated using an anti-HA antibody. Samples were analyzed by Western blot using the pSer^71^-Pfn1 antibody (F5675) or the anti-HA antibody. Densitometry was used to quantify the intensity of bands detected by F5675 and expressed as relative values to HA-Pfn1(WT). **(C)** Endogenous Pfn1 was purified from MDA-MB-231 cell lysates using poly-L-proline (PLP)-conjugated agarose beads, and subjected to Western blot analysis using the pSer^71^-Pfn1 antibody (F5675) or a pan anti-Pfn1 antibody. Input was also blotted with the pSer^71^-Pfn1 antibody (F5675) for comparison.

A search at PhosphoSitePlus revealed that Ser^71^ phosphorylation of Pfn1 has been detected in several unbiased mass spectrometry datasets (Klammer et al., 2012; Mertins et al., 2013, 2016). To confirm this, we developed a polyclonal antibody named F5675 using an antigenic Pfn1 peptide harboring pSer^71^. Using recombinant 6His-Pfn1 purified from bacteria (where serines/threonines are largely unphosphorylated), we observed by Western blot little detection of Pfn1(WT) and the phospho-resistant Pfn1(S71A) but robust detection of the phosphomimetic Pfn1(S71D) by F5675 (Figure 2A). Though chemically different from phospho-serines, phosphomimetic amino acids such as aspartate (D) or glutamate (E) have been reported by us and others to react with phospho-antibodies raised against different proteins (Shao et al., 2008b; Shu et al., 2008). This thus supports the phospho-specificity of the pSer^71^-Pfn1 antibody. Consistent with this, HA-Pfn1 immunoprecipitated from HEK293T cells was positively detected by the pSer^71^-Pfn1 antibody, and the signal was significantly reduced by the S71A mutation while enhanced by S71D (Figure 2B). However, we could not readily detect endogenous Pfn1 within whole cell lysates using the pSer^71^-Pfn1 antibody because it recognizes many other proteins due to its non-specific, polyclonal nature (Figure 2C, input, second lane). To overcome this, we used PLP-conjugated agarose beads to purify and enrich endogenous Pfn1 from the triple-negative breast cancer MDA-MB-231 cell line. This resulted in clear detection of endogenous Pfn1 by the pSer^71^-Pfn1 antibody (Figure 2C, first lane), as confirmed by the side-by-side blotting with a pan Pfn1 antibody (third lane). Thus, our data suggest that Pfn1 is phosphorylated on Ser^71^ in mammalian cells.

### Ser^71^ Is Phosphorylated by Protein Kinase A

**FIGURE 3:**
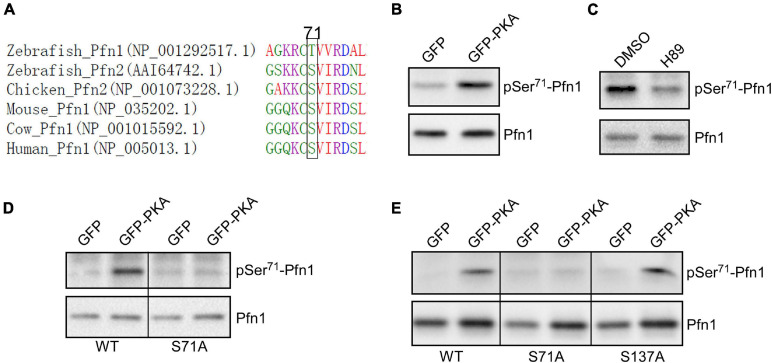
Pfn1 is phosphorylated at Ser^71^ by protein kinase A (PKA). **(A)** sequence alignment of Pfn1 and Pfn2 proteins from multiple vertebrate organisms shows that a phosphorylatable serine or threonine residue is highly conserved at position 71. **(B)** HEK293T cells were co-transfected with HA-tagged Pfn1 with GFP or GFP-PKA, followed by anti-HA immunoprecipitation. Pulldown samples were blotted using the pSer^71^-Pfn1 or pan Pfn1 antibodies. **(C)** HEK293T cells were transfected with HA-Pfn1 followed by the treatment with DMSO or 10 μM H89 for 24 h. HA-Pfn1 was subsequently immunoprecipitated and blotted as in **(B)**. **(D)** HEK293T cells were co-transfected with HA-Pfn1(WT) or HA-Pfn1(S71A) with GFP or GFP-PKA, followed by immunoprecipitation and Western blot analysis as in **(B, C)**. **(E)** HEK293T cells were co-transfected with wild type or mutant HA-Pfn1 (S71A or S137A) with GFP or GFP-PKA, followed by immunoprecipitation and Western blot analysis as in **(B–D)**.

By comparing Pfn1 and Pfn2 (a functionally related isoform) (Haarer and Brown, 1990; Witke, 2004; Jockusch et al., 2007; Birbach, 2008) sequences from various species, we find that Ser^71^ is evolutionarily conserved in vertebrates (a chemically similar threonine is present in zebrafish Pfn1) (Figure 3A). Ser^71^ is invariably preceded at −2 position by a basic residue (most commonly Arg), suggesting that it could be phosphorylated by the AGC kinase family (Pearce et al., 2010). A closer examination of the sequence surrounding Ser^71^ and comparing it with the phosphorylation consensus sites of common AGC kinases at Human Protein Kinase Knowledgebase revealed that cAMP-dependent protein kinase [also known as protein kinase A (PKA)] is a possibility. The preferred amino acids at +1 and +2 positions for PKA substrates are hydrophobic (F, I, L, and V) while those for +5 and +6 positions are leucine. The Ser^71^-containing motif in Pfn1 meets all these requirements in particular a perfect match at +5/+6 positions. To test whether PKA is indeed the kinase for Ser^71^, we co-transfected HEK293T cells with GFP or GFP-tagged alpha catalytic subunit of PKA with HA-Pfn1, and examined the level of Ser^71^ phosphorylation by Western blot after anti-HA immunoprecipitation. While equal amount of HA-Pfn1 was pulled down, co-transfection with GPKA significantly increased its pSer^71^ level detected by the F5675 antibody (Figure 3B). We next treated HEK293T cells co-transfected with HA-Pfn1 and GPKA with DMSO or PKA-specific inhibitor H89, and performed the same anti-HA immunoprecipitation and Western blot analyses. H89 significantly decreased pSer^71^-Pfn1 level without affecting the total level of HA-Pfn1 (Figure 3C). To confirm that Ser^71^, as opposed to other PKA phosphorylation sites in Pfn1, is detected by the F5675 antibody, we performed the same co-transfection experiment using either wild type or S71A mutant form of HA-Pfn1 with GFP or GPKA. We observed no GPKA-induced increase in the detection of HA-Pfn1(S71A) by the pSer^71^-Pfn1 antibody in contrast to HA-Pfn1(WT) (Figure 3D). As a separate control, mutating Ser^137^ to alanine (S137A) did not affect the ability of GPKA to increase the detection of HA-Pfn1 by the F5675 antibody (Figure 3E). Taken together, our data suggest that Ser^71^ is the site of PKA-dependent phosphorylation of Pfn1.

### Antitumor Activity of Pfn1 Requires Reversible Ser^71^ Phosphorylation and Dephosphorylation

**FIGURE 4:**
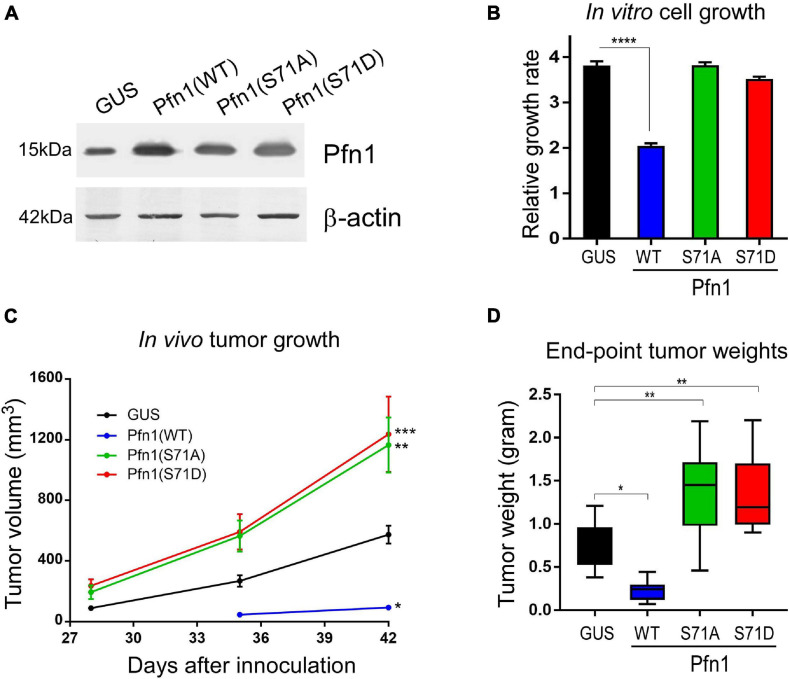
Preventing and mimicking Ser^71^ phosphorylation similarly abolish the antitumor activity of Pfn1. **(A)** Western blot analysis of exogenous wild type and mutant Pfn1 expression in stable MDA-MB-231 cells using a pan Pfn1 antibody, controlled by actin. **(B)** Stable MDA-MB-231 cells from A were seeded in 96-well plates and grown for 7 days. Relative growth rates were calculated by normalizing Alamar blue values of each subline at Day 7 to those at Day 1. Data are mean ± SEM of four technical replicates in one experiment. *P*-values were based on two-tailed unpaired *t*-test. Same results were confirmed by more than three independent experiments. **(C)** Stable MDA-MB-231 cells were injected bilaterally into the 4th mammary fat pads of 5-week-old female NOD/SCID mice (10^6^ cells per side, five mice per group). Caliper measurement of tumor volumes started at 4 weeks after injection. Data are the mean ± SEM of 10 tumors within each group. *P*-values were based on two-tailed unpaired *t*-test (versus GUS control) at day 42. **(D)** End-point tumor weights. Data are the mean ± SEM. *P*-values were based on two-tailed unpaired *t*-test (versus GUS control). *p < 0.05; **p < 0.01; ***p < 0.001, ****p < 0.0001. GUS and Pfn1(WT) tumor values were published in our prior study (Diamond et al., 2015) as they were the same controls for Pfn1(S137A vs. S137D) (published) and Pfn1(S71A vs. S71D) (shown here, unpublished) in the same mouse xenograft experiment.

Pfn1 overexpression inhibits the growth of various cancer cell lines *in vitro* and *in vivo* (Janke et al., 2000; Wittenmayer et al., 2004; Wu et al., 2006; Zou et al., 2007, 2010; Das et al., 2009; Yao et al., 2014). We have previously demonstrated that the antitumor effect of Pfn1 requires its PLP-binding ability which is inhibited by Ser^137^ phosphorylation (Diamond et al., 2015). To determine the effect of Ser^71^ phosphorylation, we virally expressed untagged wild type Pfn1 and its S71A or S71D mutants in the MDA-MB-231 breast cancer cells at levels 2–3-folds over endogenous Pfn1 (Figure 4A). GUS, a reporter gene encoding bacterial β-glucuronidase, was expressed as the negative control as previously described (Diamond et al., 2015). We first compared the cell proliferation rates *in vitro* by Alamar blue assay. While Pfn1(WT) showed anti-proliferative effect as observed previously, both Pfn1(S71A) and Pfn1(S71D) mutants were inactive (Figure 4B). We next determined the *in vivo* effects by injecting the same stable cells orthotopically in the mammary fat pads of female NOD/SCID mice as previously described (Diamond et al., 2015). Caliper measurements showed that both S71A and S71D mutations abolish the antitumor effect of Pfn1 and caused an additional increase in tumor growth compared to the GUS control (Figure 4C). End-point tumor weights confirmed these effects (Figure 4D). Thus, unlike the toggling effect of Ser^137^ phosphorylation, tumor inhibition by Pfn1 appears to require reversible Ser^71^ phosphorylation and dephosphorylation.

### Ser^71^ Phosphorylation Inhibits the Apoptosis-Sensitizing Activity of Pfn1 in Response to Paclitaxel

**FIGURE 5:**
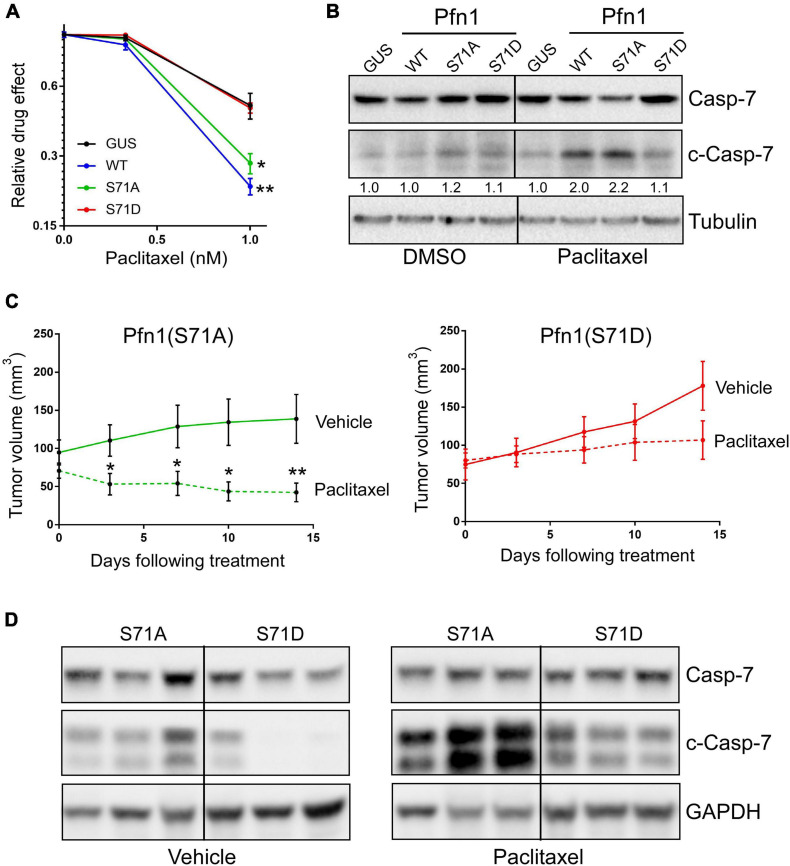
Ser^71^ phosphorylation abolishes the pro-apoptotic activity of Pfn1 in response to paclitaxel. **(A)** Stable MDA-MB-231 cells were seeded in 96-well plates and treated with DMSO or paclitaxel for 7 days. Relative drug effects were calculated by normalizing Alamar blue values of drug-treated cells by DMSO-treated cells. Data are mean ± SEM of four technical replicates in one experiment. Similar results were observed in three independent experiments. *P*-values were based on two-tailed unpaired *t*-test (versus GUS control). **(B)** Stable MDA-MB-231 cells were treated with DMSO or 5 nM paclitaxel for 48 h, followed by Western blot analysis for full-length or cleaved caspase-7 or tubulin. Cleaved caspase-7 levels were quantified by densitometry and normalized by the levels in GUS control cells in DMSO or paclitaxel groups. **(C)** MDA-MB-231 stable cells expressing Pfn1(S71A) or Pfn1(S71D) were injected bilaterally into the 4th mammary fat pads of female nude mice (10 mice per group). Tumor-bearing mice were evenly divided and treated with vehicle (0.9% sodium chloride) or paclitaxel (10 mg/kg) by intraperitoneal injection for 2 weeks. Tumor volumes were measured semiweekly by Caliper. Data are mean ± SEM. *P*-values were based on two-tailed unpaired *t*-test between vehicle and paclitaxel treatment groups at individual time points. *p < 0.05; **p < 0.01. **(D)** Randomly selected tumors harvested from vehicle or paclitaxel-treated mice in **(C)** were blotted for full-length or cleaved caspase-7 and GAPDH. Each tumor was from a different mouse.

In addition to suppressing proliferation, Pfn1 also sensitizes cancer cells to apoptosis induced by cytotoxic agents (Zou et al., 2010; Yao et al., 2013; Zaidi et al., 2016). When transfecting MDA-MB-231 cells using lipofectamine (which is cytotoxic), we consistently observed higher number of surviving cells expressing Pfn1(S71D) than those expressing Pfn1(WT) and Pfn1(S71A) (Supplementary Figure 1A). A similar effect was also observed in the transfected MCF-7 cells (Supplementary Figure 1B). Hypothesizing that Ser^71^ phosphorylation may be a pro-survival event by inhibiting the pro-apoptotic activity of Pfn1, we treated the stable MDA-MB-231 cells with paclitaxel, a commonly used chemotherapy agent which was reported to cause apoptosis in breast cancer cells more effectively upon Pfn1 overexpression (Zaidi et al., 2016). Indeed, we detected significantly decreased viability of Pfn1(WT)-expressing cells compared to the control cells. While Pfn1(S71A) showed a similar drug-sensitizing effect as Pfn1(WT), Pfn1(S71D) was completely inactive (Figure 5A). Western blot for cleaved caspase-7 confirmed the pro-apoptotic effect of Pfn1(WT) and Pfn1(S71A) but not Pfn1(S71D) upon paclitaxel treatment (Figure 5B). Similar results were also observed in MDA-MB-231 stable cells treated with doxorubicin and staurosporine (Supplementary Figure 1C,D), two other cytotoxic agents whose apoptosis-inducing abilities can be augmented by Pfn1 overexpression (Yao et al., 2013; Zaidi et al., 2016).

To confirm the toggling effect of Ser^71^ phosphorylation of Pfn1 on chemotherapy-induced apoptosis *in vivo*, we inoculated the MDA-MB-231 stable cells expressing Pfn1(S71A) vs. Pfn1(S71D) in the mammary fat pads of female nude mice. Tumors formed by both cell lines grew at similar rates as observed in the NOD/SCID mice. When the average tumor volumes in both groups reached ∼70 mm^3^, mice were randomly divided into two subgroups (*n* = 5 per group) which were treated with vehicle or paclitaxel (10 mg/kg, weekly intraperitoneal injection) for 2 weeks. Semiweekly Caliper measurement showed an obvious tumor-regressing effect of paclitaxel in the Pfn1(S71A) group, with the tumor volume difference between the vehicle and paclitaxel groups being statistically significant at the last two time points (Figure 5C). In contrast, tumors in the Pfn1(S71D) group responded much less to paclitaxel and no statistically significant difference in tumor volumes between vehicle and drug groups was detected. Western blot using tumor lysates showed significantly higher levels of cleaved caspase-7 in the paclitaxel-treated Pfn1(S71A) tumors than the Pfn1(S71D) tumors (Figure 5D). Interestingly, baseline cleaved caspase-7 levels in untreated tumors, though much lower than in paclitaxel-treated tumors, were also higher in the Pfn1(S71A) vs. Pfn1(S71D) tumors. Collectively, these data suggest that Ser^71^ phosphorylation, by blocking actin-binding of Pfn1, abolishes the apoptosis-sensitizing activity of Pfn1 particularly in response to chemotherapy agents such as paclitaxel.

### Apoptotic Sensitization by Pfn1 Rrequires Actin-Binding and Cytoplasmic Localization

**FIGURE 6:**
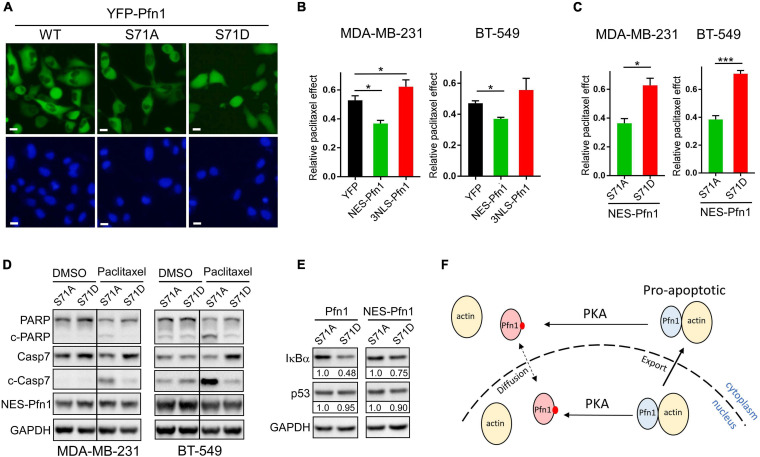
Apoptotic sensitization by Pfn1 requires actin-binding and cytoplasmic localization. **(A)** Direct fluorescence imaging of MDA-MB-231 stably infected with wild type or mutant YFP-Pfn1. Scale bars represent 20 μm. **(B)** Stable MDA-MB-231 and BT-549 cells expressing YFP, NES-Pfn1, or NLS-Pfn1 were subjected to colony formation assays in 24-well plates with vehicle (DMSO) or 0.5 nM paclitaxel for 7 days. Relative drug effects were calculated as the ratios between cell numbers (based on Alamar blue values) in the paclitaxel-treated vs. vehicle conditions. **(C)** Stable MDA-MB-231 and BT-549 cells expressing NES-Pfn1(S71A) or NES-Pfn1(S71D) were treated with vehicle or paclitaxel in colony formation assays and analyzed as in **(B)**. Data are mean ± SEM of three technical replicates in one biological experiment. Similar results were observed in three independent experiments. *P*-values were based on two-tailed unpaired *t*-test. *p < 0.05; ***p < 0.001. **(D)** MDA-MB-231 and BT-549 stable cells expressing NES-Pfn1(S71A) or NES-Pfn1(S71D) were treated with DMSO or 5 nM paclitaxel for 48 h, and analyzed for full-length or cleaved PARP and caspase-7, YFP-NES-Pfn1 (with GFP ab) and GAPDH by Western blot. **(E)** MDA-MB-231 stable cells expressing untagged or NES-tagged Pfn1(S71A) vs. Pfn1(S71D) were treated with 10 nM paclitaxel for 48 h, and analyzed for IκBα, p53, and GAPDH by Western blot. **(F)** Hypothetical model for the pro-apoptotic activity of cytoplasmic Pfn1 which requires actin-binding and is abolished by PKA-dependent Ser^71^ phosphorylation. Ser^71^ may also be phosphorylated by PKA in the nucleus. Actin-free pSer^71^-Pfn1, due to its small size, can freely diffuse in and out of nucleus. However, nuclear pSer^71^-Pfn1 cannot be actively transported into the cytoplasm by exportin-6 due to its loss of actin-binding.

The ability of Pfn1 to bind actin is crucial for its nuclear export by exportin-6 (Stuven et al., 2003). Since Ser^71^ phosphorylation disrupts actin-binding of Pfn1, we examined its effect on Pfn1 subcellular localization. Upon transfection into HEK293T cells, YFP-tagged Pfn1(WT) and Pfn1(S71A) were predominantly localized in the cytoplasm. However, YFP-Pfn1(S71D) was diffusely present within cytoplasm and nucleus (Figure 2A). We observed the same phenotype using lentivirus-infected MDA-MB-231 cells (Figure 6A). The opposite effects of S71A vs. S71D mutations on cell survival upon paclitaxel treatment were observed in the context of YFP-Pfn1 similar to untagged Pfn1 (Supplementary Figure 2B). Intrigued by the effects of Ser^71^ phosphorylation on both Pfn1 subcellular localization and chemo-sensitizing activity, we tested whether these two phenotypes are causally linked. To do that, we forced YFP-Pfn1 expression either in the cytoplasm or nucleus by tagging it with a nuclear export sequence (NES, recognized by exportin-1) or nuclear localization sequence (NLS) as previously described (Diamond et al., 2015; Zhu et al., 2021). We virally introduced YFP, YFP-NES-Pfn1, or YFP-NLS-Pfn1 into MDA-MB-231 cells and determined their relative responses to paclitaxel treatment. Compared to YFP control cells, YFP-NES-Pfn1 cells showed higher sensitivity to paclitaxel treatment while YFP-NLS-Pfn1 cells showed less (Figure 6B). These opposite effects on cellular survival by YFP-NES-Pfn1 and YFP-NLS-Pfn1 were also observed in BT-549 cells, another triple-negative breast cancer cell line (Figure 6B).

Next, we examined whether the drug-sensitizing effect of NES-Pfn1 is still under the regulation of Ser^71^ phosphorylation by introducing the S71A vs. S71D mutations (Supplementary Figure 2C). Indeed, MDA-MB-231 and BT-549 cells expressing NES-Pfn1(S71D) showed significantly higher resistance to paclitaxel treatment than those expressing NES-Pfn1(S71A) in colony formation assays (Figure 6C). Western blot against cleaved caspase-7 and cleaved PARP confirmed that paclitaxel-induced apoptosis in NES-Pfn1(S71D)-expressing cells was indeed significantly lower than in NES-Pfn1(S71A)-expressing cells (Figure 6D).

The pro-apoptotic activity of Pfn1 has been causally linked to its ability to increase p53 and IκBα (negative regulator of NFκB) levels in breast cancer cell lines (Yao et al., 2013; Zaidi and Manna, 2016; Zaidi et al., 2016). We found that MDA-MB-231 stable cells expressing untagged Pfn1(S71D) contain significantly lower levels of IκBα than those expressing Pfn1(S71A) (Figure 6E). The difference in IκBα levels between MDA-MB-231 cells expressing NES-Pfn1(S71A) and NES-Pfn1(S71D) can also be seen but to a lesser extent (Figure 6E). Interestingly, p53 levels did not differ significantly between Pfn1(S71A) and Pfn1(S71D) cells regardless of whether Pfn1 is untagged or NES-tagged (Figure 6E). Collectively, our data suggest that cytoplasmic Pfn1 promotes apoptosis in response to cytotoxic treatments and such an activity is abolished by Ser^71^ phosphorylation via abolishing actin-binding of Pfn1 as well as causing its nuclear retention, both of which contribute to IκBα destabilization and increased NFκB signaling.

## Discussion

Pfn1 was the first actin-binding protein identified more than four decades ago (Carlsson et al., 1977), yet it remains uncertain to this day whether its actin-binding ability undergoes negative regulation by post-translational modifications. In this paper, through candidate mutagenesis and validation using public proteomic data and a custom-made phospho-specific antibody, we provided evidence that the evolutionarily conserved Ser^71^ is a *bona fide* phosphorylation site in Pfn1 selectively inhibiting its actin-binding with little effect on its PLP-binding. This is consistent with the fact that Ser^71^ is located within the actin-binding site of Pfn1 and distal to its PLP-binding pocket (Ferron et al., 2007). Sequence analysis combined with genetic and pharmacological testing suggested that PKA may be one of the kinases phosphorylating Ser^71^. Interestingly, it was recently suggested that PKA can also phosphorylate Ser^137^ of Pfn1 (Gau et al., 2019), a shared target site for PKC and ROCK which specifically inhibits Pfn1 interaction with PLPs (Singh et al., 1996; Sathish et al., 2004; Shao et al., 2008a). Nonetheless, based on co-immunoprecipitation data using HA-Pfn1-transfected HEK293T cells, the stoichiometry of PKA-mediated Ser^71^ phosphorylation of total cellular pool of Pfn1 appeared low, as overexpression of the alpha catalytic subunit of PKA did not cause detectable reduction in the levels of associated actin (data not shown), despite increasing Ser^71^ phosphorylation levels. This could be due to several possible reasons. First, PKA-mediated Ser^71^ phosphorylation may occur to a small fraction of total cellular Pfn1 either transiently or at specific subcellular locations. Given the high cellular abundance of Pfn1 (>50 μM in most tissues) (Witke, 2004), the net effect on steady-state actin-binding would not be detectable by co-IP using whole cell lysates. Second, PKA activity is regulated by complex mechanisms including inhibitory binding of the regulatory subunits as well as tethering to specific subcellular locations via the diverse A-kinase-anchoring proteins (AKAP) (Francis and Corbin, 1994; Harada et al., 1999). Therefore, it is possible that overexpressing the catalytic subunit of PKA alone is insufficient to achieve optimal Pfn1 phosphorylation. In either case, more molecular details regarding the newly identified PKA/pSer^71^-Pfn1 axis and the possible involvement of other kinases remain to be determined in the future.

Despite being an essential actin-binding protein, Pfn1 simultaneously functions as a non-classical tumor suppressor across different malignancies including breast (Roy and Jacobson, 2004; Wittenmayer et al., 2004; Zou et al., 2007, 2009, 2010; Bae et al., 2009, 2010; Das et al., 2009; Diamond et al., 2015), pancreatic (Yao et al., 2014), and liver (Wu et al., 2006) cancers. Its ability to inhibit cell cycle progression and tumor cell growth has been demonstrated by many *in vitro* and *in vivo* studies. Our recent work suggested that tumor growth inhibition by Pfn1 is mediated at least in part by its nuclear function in repressing SEC-dependent transcription of pro-cancer genes including c-MYC (Diamond et al., 2015; Zhu et al., 2021). Such an activity can be toggled off and on by Ser^137^ phosphorylation and dephosphorylation (mimicked by S137D and S137A) which, respectively, blocks and enables Pfn1 binding to PLPs that are present in the SEC component ENL (Diamond et al., 2015; Zhu et al., 2021). In this paper, by analyzing the effects of pSer^71^-resistant and mimetic mutants (S71A and S71D) in MDA-MB-231 cells, we made the unexpected observation that both cannot inhibit tumor growth. This indicates that, rather than functioning as an on/off switch, reversible phosphorylation and dephosphorylation at Ser^71^, causing dynamic actin dissociation and rebinding, are required for Pfn1 to inhibit tumor growth. Given that actin-binding promotes nuclear export of Pfn1 (Stuven et al., 2003), this raises an interesting possibility that Ser^71^ phosphorylation may occur in a transient and tightly controlled manner to nuclear Pfn1 to allow its escape from nuclear export, which is subsequently followed by Ser^71^ dephosphorylation to render actin-rebinding by Pfn1 for its functional engagement in transcriptional repression. Though speculative at the moment, this theory is consistent with our current knowledge regarding Pfn1 functions and can be tested in the future.

In addition to suppressing tumor growth, Pfn1 also sensitizes cancer cells to drug-induced apoptosis. This has been best demonstrated in breast cancer cells treated with cytotoxic agents many of which are common cancer chemotherapies including paclitaxel (Zou et al., 2010; Yao et al., 2013; Zaidi et al., 2016). Although detailed molecular mechanisms underlying apoptosis-sensitization by Pfn1 remain unknown, such an activity has been linked to p53 and NFκB signaling. It was found that Pfn1 and p53 co-exist in the same complex in breast cancer cells, and Pfn1 overexpression increases total p53 protein levels as well as its redistribution to cytoplasm and mitochondria (Yao et al., 2013; Zaidi et al., 2016) where p53 can drive intrinsic apoptotic pathway in a transcription-independent fashion (Marchenko et al., 2000; Chipuk et al., 2004). In support of the functional link between Pfn1 and p53, apoptosis-sensitizing effect of Pfn1 was greatly reduced in p53-null and knockdown cells (Yao et al., 2013; Zaidi et al., 2016). In addition to enhancing p53 activity, Pfn1 was also found to decrease NFκB signaling by preventing cytotoxin-induced IκBα phosphorylation and degradation and consequently preventing p65 nuclear translocation and transcription of pro-survival genes (Zaidi and Manna, 2016; Zaidi et al., 2016). Although these studies implicate the involvement of cytoplasmic Pfn1 in apoptotic sensitization, direct evidence was unavailable. In this study, by tagging Pfn1 with NES or NLS, we showed that apoptotic sensitization by Pfn1 indeed requires its cytoplasmic localization. Interestingly, such an activity of Pfn1 can be switched off and on by pSer^71^-Pfn1 mimicking and preventing mutants both *in vitro* and *in vivo*, indicating an essential role of actin-binding. Our data suggested that pSer^71^-Pfn1 could be a novel predictive biomarker for cancer chemotherapy response. They are also consistent with the well-known pro-survival effects of PKA, which have been mechanistically linked to activating phosphorylation of the cAMP-response element binding protein (CREB) in the nucleus (Wilson et al., 1996; De Cesare and Sassone-Corsi, 2000; Mayr and Montminy, 2001; Naqvi et al., 2014) and deactivating phosphorylation of the pro-apoptotic protein Bad in the cytoplasm (Harada et al., 1999; Lizcano et al., 2000). Thus, Pfn1 may be a previously unknown downstream effector of PKA function in apoptotic inhibition through Ser^71^ phosphorylation. Given that Ser^71^ phosphorylation prevents nuclear Pfn1 export, we speculate that it may inhibit the apoptosis-sensitizing effect of Pfn1 via a two-pronged mechanism by abolishing its actin-binding and reducing its cytoplasmic levels (Figure 6F). Taken together, our data in this paper demonstrated that Ser^71^ is a *bona fide* phosphorylation site of Pfn1 capable of inhibiting its actin-binding ability, preventing its nuclear export, and influencing its tumor-inhibitory functions.

## Data Availability Statement

The raw data supporting the conclusions of this article will be made available by the authors, without undue reservation.

## Ethics Statement

The animal study was reviewed and approved by The Animal Studies Committee at Washington University in St. Louis.

## Author Contributions

JS conceived of the project and wrote the manuscript with input from all authors. JS, FW, CZ, and S-JK performed the experiments. JS, FW, CZ, SC, and S-JK performed the experiments and analyzed the data. AB and MB provided technical expertise and intellectual input.

## Conflict of Interest

The authors declare that the research was conducted in the absence of any commercial or financial relationships that could be construed as a potential conflict of interest.
