# Supplementary material for: Ser71 Phosphorylation Inhibits Actin-Binding of Profilin-1 and Its Apoptosis-Sensitizing Activity
Source: Front Cell Dev Biol. 2021 Jun 21;9:692269. doi: 10.3389/fcell.2021.692269 (PMC8255618; doi:10.3389/fcell.2021.692269)
Supplement: Supplementary file 1 [file Data_Sheet_1.pdf]

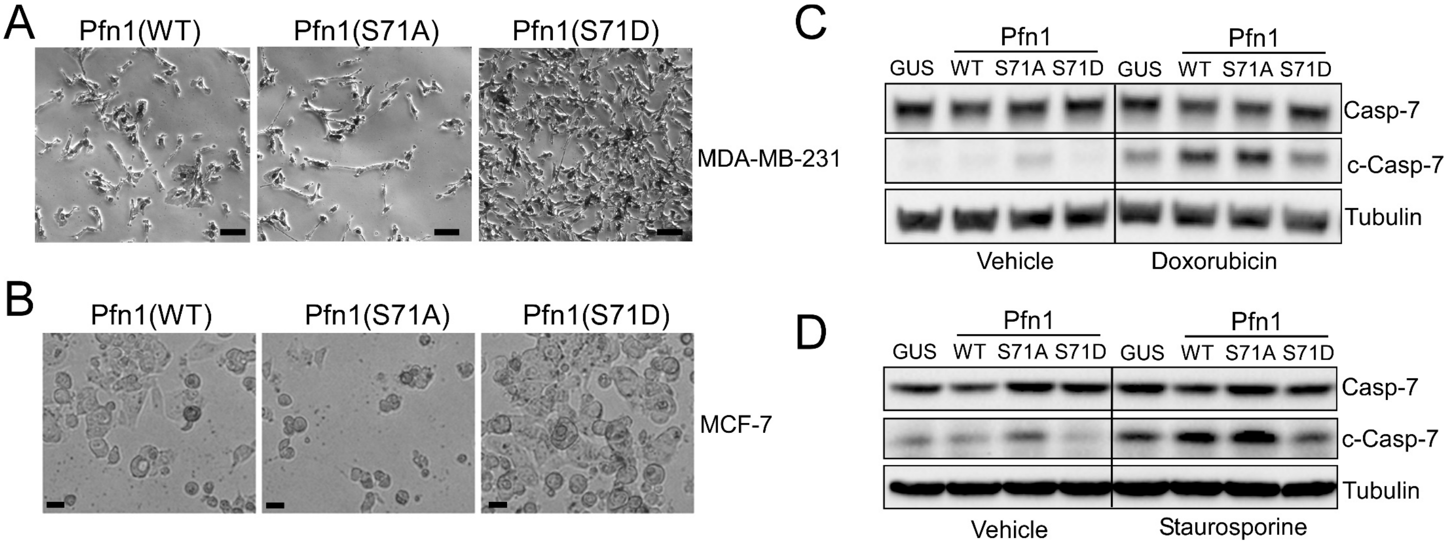

Figure S1. Ser^{71} phosphorylation inhibits the pro-apoptotic activity of Pfn1 in response to different stimuli, related to Fig.5. ***A-B*,** Same numbers of MDA-MB-231 and MCF-7 cells were transiently transfected with untagged wild type or mutant Pfn1 by lipofectamine 2000. Bright-field cell images were acquired three days after transfection. ***C-D***, MDA-MB-231 stable cells expressing GUS or untagged wild type or mutant Pfn1 were treated with 300nM doxorubicin for 48 hours (*C*) or 20nM staurosporine for 8 hours (*D*). Lysates were blotted for full-length and cleaved caspase-7 and tubulin.

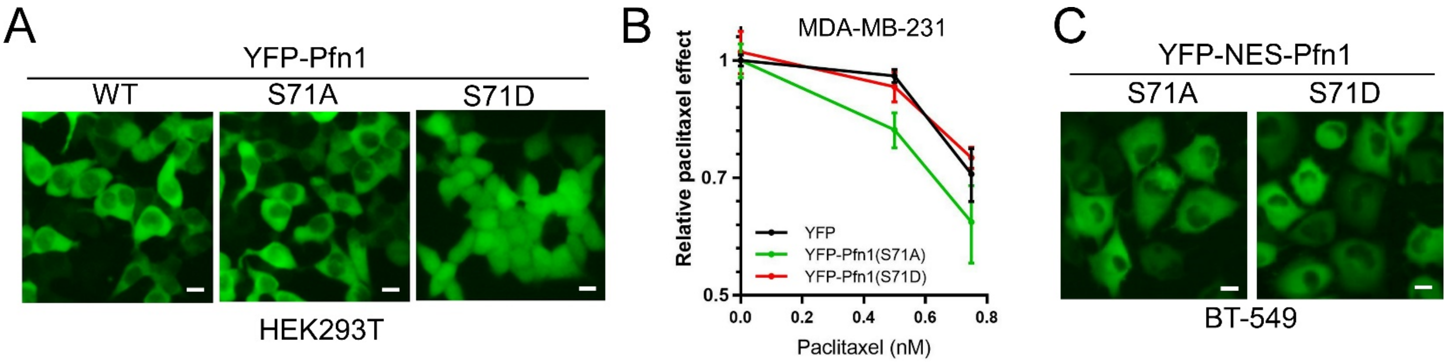

Figure S2. Subcellular localization of wild type and mutant YFP-Pfn1 and their effects on the survival of paclitaxel-treated cells, related to Fig.6. ***A***, Direct fluorescence imaging of HEK293T cells infected with lentiviruses expressing YFP-tagged wild type or mutant Pfn1. ***B***, MDA-MB-231 stable cells expressing YFP, YFP-Pfn1(S71A), or YFP-Pfn1(S71D) were subjected to colony formation assays in the presence of vehicle or paclitaxel for 5 days. Relative drug effects were calculated as in Fig.5A. ***C***, Direct fluorescence imaging of BT-549 cells stably expressing YFP-NES-Pfn1 containing S71A or S71D mutations. Scales bar represent 20μm.
